# Epidemiology of posttraumatic stress disorder: A prospective cohort study based on multiple nationwide Swedish registers of 4.6 million people

**DOI:** 10.1192/j.eurpsy.2022.2311

**Published:** 2022-09-08

**Authors:** Syed Rahman, Stanley Zammit, Christina Dalman, Anna-Clara Hollander

**Affiliations:** 1 Epidemiology of Psychiatric Conditions, Substance use and Social environment (EPICSS), Department of Global Public Health, Karolinska Institutet, Stockholm, Sweden; 2 Division of Insurance Medicine, Department of Clinical Neuroscience, Karolinska Institutet, Stockholm, Sweden; 3 Division of Psychological Medicine and Clinical Neurosciences, Cardiff University, Cardiff, United Kingdom; 4 Centre for Academic Mental Health, Population Health Sciences, Bristol Medical School, Bristol, United Kingdom; 5 Centre for Epidemiology and Community Medicine, Region Stockholm, Stockholm, Sweden

**Keywords:** Anxiety disorders, common mental disorders, epidemiology, posttraumatic stress disorder, transcultural psychiatry

## Abstract

**Background:**

Experiencing exceptionally threatening or horrifying traumas can lead to posttraumatic stress disorder (PTSD). Increasing political unrest/war/natural disasters worldwide could cause more traumatic events and change the population burden of PTSD. Most PTSD research is based on surveys, prone to selection/recall biases with inconsistent results. The aim was therefore, to use register-based data to identify the occurrence of PTSD and contributing factors in the Swedish general population.

**Methods:**

This register-based cohort study used survival analysis. Individuals born between 1960–1995, aged ≥15 years, registered and living in Sweden, not emigrating, anytime between 1990–2015, not receiving specialized care for PTSD before 2006 were included (*N* = 4,673,764), and followed from their 15th/16th birth date until first PTSD diagnosis between 2006–2016 or study endpoint (31-December-2016). PTSD cases (ICD-10: F43.1) were identified from the national patient register. Mean follow-up time was 18.8 years.

**Results:**

Between 2006–2016, the incidence of specialized healthcare utilization for PTSD nearly doubled, and 0.7% of the study population received such care. The highest risk was observed for refugees [aHR 8.18; 95% CI:7.85–8.51] and for those with depressive disorder [aHR 4.51; 95% CI:3.95–5.14]. Higher PTSD risk was associated with female sex, older age, low education, single parenthood, low household income, urbanicity, and being born to a foreign-born parent.

**Conclusions:**

PTSD is more common among refugee migrants, individuals with psychiatric disorders, and the socioeconomically disadvantaged. It is important that provision of services for PTSD are made available, particularly to these higher risk, and often hard-to-reach groups.

## Introduction

Posttraumatic stress disorder (PTSD) is a psychiatric disorder that can develop after a person has been exposed to exceptionally threatening or horrifying events [1], for example, threatened death, serious injury, or sexual violation [1]. Many people experience traumas without developing PTSD [1], however, specific risk factors for developing PTSD after a trauma include both number of traumas and consequences of the trauma (e.g., pain) and quality of social support [2]. PSTD exists all over the world and there is a strong case for its cross-cultural validity [1]. Still, estimates of PTSD prevalence differ between countries. In one large study, Dückers et al. [3] used the Composite International Diagnostic Interview, to compare data from 24 countries, and reported lifetime PTSD prevalence in the general population ranging from 0.3 to 9.2% (mean 3.2%). A European study, using World Mental Health (WMH) survey data from 11 countries, reported PTSD prevalence between 0.38 and 6.67% [4]. In Sweden in 2005, Frans et al. [5] found a 5.6% lifetime prevalence of PTSD. All these studies reporting country estimates of PTSD were based on surveys with a large proportion of non-responders, with an average of 29% in the Dückers et al. [3] study, and nearly 40% in the Frans et al. [5] study.

In Sweden, all visits to healthcare are recorded in local and national administrative registers covering the entire population, which has greatly benefited mental health research [6]. To our knowledge, no study has so far used register-based methods to estimate the national prevalence or incidence of PTSD.

The study aimed to estimate the incidence of diagnosed PTSD in Sweden and to identify the possible association between demographic factors (sex, age, education, household income, family composition, living area, migration status) and health related (psychiatric and major somatic morbidity) factors, with subsequent development of PTSD.

## Methods

### Data sources

This is a prospective cohort study based on multiple nationwide Swedish registers from the register linkage “Psychiatry Sweden” [7]. Data from different registers were merged using unique de-identified personal identification numbers assigned to all individuals born or registered to live in Sweden [8], excluding those without a residence permit such as asylum seekers. The study used information from the Total Population Register [9], longitudinal integrated database for health insurance and labor market studies (LISA) [10], longitudinal database for integration studies (STATIV) [11], National Patient Register (NPR) [12], Causes of Death Register [13] and the Multigeneration Register [14].

### Study population

All individuals born between 1960 and 1995, aged 15 years or older, registered and living in Sweden anytime between 1990 and 2015 (*N* = 4,674,960) were included. Thereafter, individuals emigrating from Sweden (*n* = 923) or having been treated for PTSD at an inpatient or specialized outpatient care before 2006 (*n* = 273) were removed. Thus, the final study population comprised 4,673,764 individuals.

### PTSD

PTSD in this study is conceptualized as receiving treatment at an inpatient or specialized outpatient care due to PTSD, henceforth referred to as treatment for PTSD. Patients were identified from the NPR by the codes of International Classification of Diseases 10th revision (ICD-10) for PTSD F43.1. Due to the quality of the PTSD diagnosis in the NPR, we considered only those diagnosed between 2006 and 2016. The Swedish National Patient Register is considered of good validity [12], and recently the PTSD diagnoses in the NPR were validated and found feasible to use in research [15].

### Covariates

Sociodemographic factors were measured on 31st December of the year preceding the start of follow-up. They included sex, age, education, living area, weighted household disposable income, family composition, parental country of birth, and migration status. Migration status was divided into four categories: Swedish-born with Swedish-born parents, refugee migrants, non-refugee migrants, and second-generation migrants. Refugee migrants were defined according to the Geneva Convention of Refugees [16], or if someone was granted a residence permit based on “humanitarian ground” and “in need of protection” [17]. Non-refugee migrants are those born abroad, with at least one parent born abroad, and who later settled in Sweden for work, family reunion, study, investments, and so forth. Second-generation migrants were defined as being born in Sweden with at least one parent born abroad.

Comorbid psychiatric and somatic diagnoses were included in the medical factors and measured before the start of follow-up (since 1987 for inpatient and 2001 for outpatient). All diagnoses were extracted from the NPR and defined according to ICD-10. The comorbid psychiatric disorders included non-affective psychosis (NAP) (F20–F29), depressive disorders (F32–F33), anxiety and stress-related disorders (F40–F43, except F43.1), alcohol abuse (F10, G31.2, G62.1, G72.1, I42.6, K29.2, K70, K86.0, O35.4, P04.3, Q86.0, T51.0, X45, Y91, Z50.2, Z71.4), suicide attempt (X60–X84, Y10–Y34), and other comorbid psychiatric disorders (any “F” code except the above mentioned); the somatic incorporated diabetes (DM) (E10–E14), circulatory (I00–I99), respiratory (J00–J99) and musculoskeletal disorders (M00–M99). All diagnoses in this study were dichotomized as “yes/no”.

### Swedish healthcare system

Sweden has a “need-based” decentralized healthcare system with universal insurance coverage [18]. All residents have equal and universal healthcare access based on their health need. The system is hugely subsidized by the government and restricted by a high-cost ceiling. Maximum annual out-of-pocket costs for medical consultations and prescription drugs are 125 USD and 255 USD, respectively, with free medical services for people under 18 years.

### Statistical analysis

Differences in the distribution of covariates across the population groups were determined by chi-squared tests, thereafter we applied Cox’s proportional hazard models to ascertain the risk indicators for specialized healthcare utilization due to PTSD in the Swedish general population, including all individuals aged between 15/16 and 64 years, registered and living in Sweden anytime between 1990 and 2015. The four cox models included the following: model 1: unadjusted/crude model; model 2: adjusted for sex, age, education, family composition, weighted household income, living area, parental country of birth, and migration status; model 3: additionally adjusted for different psychiatric disorders; model 4: full adjusted model—additionally adjusted for major somatic disorders that are mentioned in the table. Each individual was followed up from the age of 15 (born in 2010 or later) or 16 (born before 2010) years. At this age people in Sweden become enrolled in the LISA register, containing socioeconomic information of the entire population. In the case of migrants registering in Sweden at a later age, the incident year of registration in LISA was considered as the start follow-up year. Individuals were followed up until registered care for PTSD at a specialized care facility between 2006–2016 or the study endpoint, i.e., 31 December 2016. Censoring was applied for death or emigration during the follow-up.

All analyses were performed using the statistical software SAS v. 9.4.

## Ethical Approval

This study belongs to Psychiatry Sweden “Mental health, psychiatric disorders: occurrence and aetiology” project, and the analysis of this study, based on different Swedish national registers, has been approved by the Stockholm Regional Ethical Review Board (number 2010/1185–31/5). In Sweden, ethical vetting is always required when using registered data and performed by regional review boards. The different registers used for this study were anonymized and de-identified prior to analysis by Statistics Sweden, which was responsible for data linkage. The researchers received de-identified data.

### Results

Our study cohort comprised 4,673,764 individuals from the whole of Sweden, including 31,608 (0.7%) with incident specialized care treatment for PTSD between 2006 and 2016 (Table 1). The study population included an approximately even sex distribution (male 51% vs. female 49%), however, among those who eventually received treatment for PTSD, the proportion of females was double that of males (66% vs. 34%). The majority were followed up from age 15 or 16, consequently, the cohort was dominated by the youngest age group 15–19 years (60%), with low education (>10 years) (58%), and mostly living with parents (62%). About half of the study population (>51%) belonged to the lowest household income quintile, whereas among the PTSD population more than 71% belonged to the lowest income quintile. More than two-third of the cohort were born to Swedish-born parents, while around 57% of the PTSD population were either born to at least one foreign-born parent or did not have information regarding parental birth country. Additionally, refugees and non-refugee migrants were more common among the PTSD population than non-PTSD (32 and 11% vs. 18 and 2%, respectively).

**Table 1.** Descriptive statistics of the study population (*N* = 4,673,764).

	All (*N* = 4,673 764) *n* (%)	PTSD (*n* = 31,608) *n* (%)	No PTSD (*n* = 4,642,156) *n* (%)	*χ*^2^ for differences (*p*-value)^b^
Sex				<0.0001
Male	2,392,411 (51.19)	10,609 (33.56)	2,381,802 (51.31)	
Female	2,281,353 (48.81)	20,999 (66.44)	2,260,354 (48.69)	
Age (in years)				<0.0001
15–19	2,804,880 (60.01)	17,490 (55.33)	2,787,390 (60.05)	
20–29	1,503,955 (32.18)	9,048 (28.63)	1,494,907 (32.20)	
30–39	309,488 (6.62)	3,963 (12.54)	305,525 (6.58)	
40–49	53,920 (1.15)	1,087 (3.44)	52,833 (1.14)	
50–59	1,521 (0.03)	20 (0.06)	1,501 (0.03)	
Education (in years)				<0.0001
0–9 (school)	2,675,304 (57.63)	52,488 (55.70)	2,640,832 (57.67)	
10–12 (college)	1,080,581 (23.28)	26,200 (27.80)	1,060,531 (23.16)	
≥13 (higher)	413,960 (8.92)	7,737 (8.21)	408,374 (8.92)	
Missing	472,311 (10.21)	7,814 (8.29)	469,189 (10.25)	
Family composition				<0.0001
With partner no child	165,411 (3.54)	1,422 (4.5)	163,989 (3.53)	
With partner and child	482,446 (10.32)	5,116 (16.19)	477,330 (10.28)	
Single^a^ no child	996,973 (21.33)	5,645 (17.86)	991,328 (21.35)	
Single with child	120,746 (2.58)	1,719 (5.44)	119,027 (2.56)	
With parents, aged <20	2,908,042 (62.22)	17,701 (56.0)	2,890 341 (62.26)	
Missing	2,146 (0.00)	5 (0.02)	141 (0.00)	
Household income				<0.0001
First quintile (lowest)	2,389,260 (51.12)	22,489 (71.15)	2,366,771 (50.98)	
Second quintile	803,995 (17.20)	3,957 (12.52)	800,038 (17.23)	
Third quintile	617,414 (13.21)	2,614 (8.27)	614,800 (13.24)	
Fourth quintile	497,480 (10.64)	1,634 (5.17)	495,846 (10.68)	
Fifth quintile (highest)	365,487 (7.82)	910 (2.88)	364,577 (7.85)	
Missing	128 (0.00)	4 (0.01)	124 (0.00)	
Living area^b^				<0.0001
Large cities	1,596,470 (34.16)	11,777 (37.26)	1,584,693 (34.14)	
Medium-sized towns	1,805,013 (38.62)	11,687 (36.97)	1,793,326 (38.63)	
Small towns/rural area	1,272,221 (27.22)	8,142 (25.76)	1,264 079 (27.23)	
Missing	60 (0.0)	2 (0.1)	58 (0.0)	
Mother’s country of birth				<0.0001
Sweden	3,326,364 (71.17)	15,360 (48.6)	3,311,004 (71.32)	
Outside of Sweden	626,151 (13.4)	5,573 (17.63)	620,578 (13.37)	
Missing	721,249 (15.43)	10,675 (33.77)	710,574 (15.31)	
Father’s country of birth				<0.0001
Sweden	3,280,880 (70.20)	14,759 (46.69)	3,266,121 (70.36)	
Outside of Sweden	590,678 (12.64)	5,256 (16.63)	585,422 (12.61)	
Missing	802,206 (17.16)	11,593 (36.68)	790,613 (17.03)	
Parental country of birth				<0.0001
Both born in Sweden	3,101,054 (66.35)	13,653 (43.19)	3,087,401 (66.51)	
One/both born outside Sweden	867,558 (18.56)	7,507 (23.75)	860,051 (18.53)	
Missing	705,152 (15.09)	10,448 (33.05)	694,704 (14.97)	
Migration status				<0.0001
Non-migrants	3,101,054 (66.35)	13,653 (43.19)	3,087,401 (66.51)	
Second-generation migrants	602,300 (12.89)	4,307 (13.63)	597,993 (12.88)	
Non-refugee migrants	862,112 (18.45)	10,040 (31.76)	852,072 (18.36)	
Refugee migrants	108,298 (2.32)	3,608 (11.41)	104,690 (2.26)	
Psychiatric morbidity at baseline (only “yes”)				
Non-affective psychosis	404 (0.01)	28 (0.09)	376 (0.01)	<0.0001
Depressive disorders	6,097 (0.13)	369 (1.17)	5,728 (0.12)	<0.0001
Anxiety disorders	8,405 (0.18)	406 (1.28)	7,999 (0.17)	<0.0001
Alcohol use disorders	8,165 (0.17)	181 (0.57)	7,984 (0.17)	<0.0001
Other mental disorders	44,739 (0.96)	775 (2.45)	43,964 (0.95)	<0.0001
Suicide attempt	893 (0.02)	78 (0.25)	815 (0.02)	<0.0001
Somatic comorbidity (only “yes”)				
Circulatory disorders	12,360 (0.26)	128 (0.4)	12,232 (0.26)	<0.0001
Respiratory disorders	152,619 (3.27)	1,281 (4.05)	151,338 (3.26)	<0.0001
Diabetes	7,577 (0.16)	71 (0.22)	7,506 (0.16)	0.0056
Musculoskeletal disorders	96,024 (2.05)	912 (2.89)	95,112 (2.05)	<0.0001

Abbreviation: PTSD, posttraumatic stress disorder.

a Single includes divorced, separated, or widowed.

b Large cities: municipalities with at least 200,000 inhabitants with at least 200,000 in the largest urban area (Stockholm, Gothenburg and Malmo); Medium-sized cities: municipalities with at least 50,000 inhabitants with at least 40,000 in the largest urban area; Small cities/villages: municipalities with at least 15,000 inhabitants in the largest urban area.

The psychiatric profile of the PTSD-population was much worse than the non-PTSD with substantially higher comorbid depression (1.2 vs. 0.1%), anxiety disorders (1.3 vs. 0.2%), NAP (0.09 vs. 0.01), alcohol use disorders (0.6 vs. 0.2%), and suicide attempt (0.3% vs. 0.02%). Somatic comorbidity was also higher among the PTSD population compared to the non-PTSD, though to a lesser extent than psychiatric comorbidity (Table 1).

**Figure 1.**
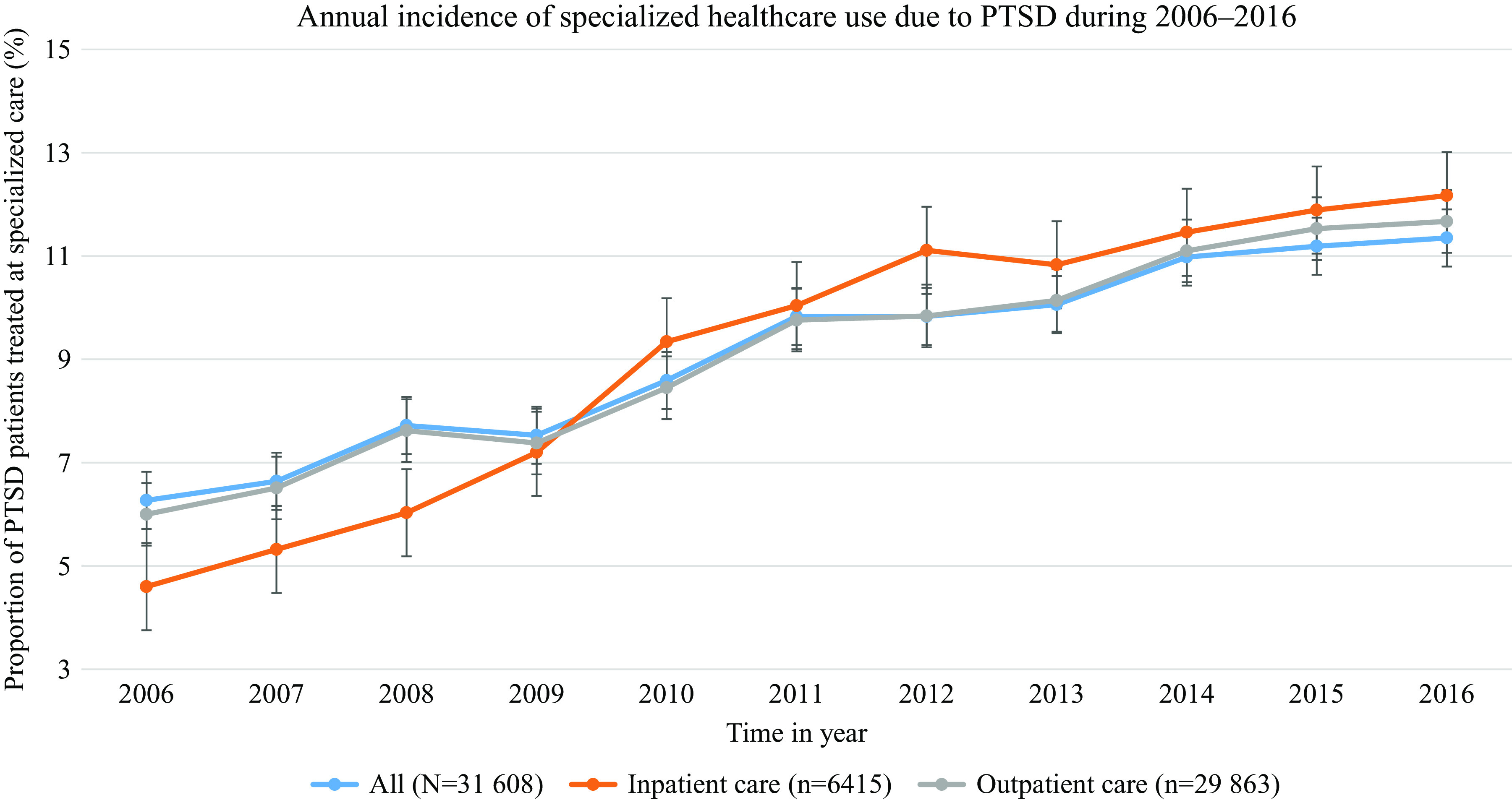
Annual incidence of specialized healthcare (in- and outpatient) use for PTSD among the PTSD patients (*n* = 31,608) in the study population in Sweden.

We observed a steady increase in the incidence of specialized care treatment for PTSD in the Swedish general population between 2006 and 2016, nearly doubling in the span of 10 years. Among the total PTSD patients receiving incident specialized care (*n* = 31,608; 0.7% of the total study population *N* = 4,673,764), 6.27% (*n* = 1,983) received incident specialized care in 2006, whereas in 2016 proportion of such patients was 11.35% (3,586). Stratified analysis showed a similar picture for psychiatric inpatient and specialized outpatient care (Figure 1).

Table 2 shows the hazard ratios (HRs) with 95% confidence intervals (CIs) for subsequent PTSD in the study population. Women had a two-fold higher risk of specialized care for PTSD compared to men [aHR 2.0; 95% CI:1.96–2.05]. We observed a positive dose–response relationship between age and PTSD starting from age 30 [range of aHRs 1.29–3.09]. Noticeably, the age group 20–29 had a lower risk of specialized care for PTSD diagnosis, treated at specialized healthcare [aHR 0.75; 95% CI:0.7–0.8] compared to the youngest group 15–19 years. Such PTSD risk was lower with higher education; individuals with university education (≥13 years) had the lowest PTSD risk compared to those with mandatory school education (9 years) [aHR 0.57; 95% CI:0.55–0.6]. A similar association was observed regarding weighted household income; the lower the income the greater the risk for subsequent specialized care for PTSD [lowest income group vs. the highest: aHR 2.93; 95% CI: 2.73–3.14]. Regarding family composition, being a single parent was associated with increased risk [aHR 1.44; 95% CI:1.34–1.54] compared to those with a partner and no child living in the household. Living area was also significantly associated with subsequent risk of PTSD diagnosis, with a reduced risk for inhabitants of medium-sized or small towns/villages compared to large city dwellers [range of aHRs 0.91–0.93]. Our crude models suggested that having foreign-born parent/parents was associated with a greater than two-fold increase in specialized care for PTSD [HR 2.25; 95% CI:2.19–2.32]. Due to collinearity between parental birth country and migration status, this variable was not included in the adjusted model. Migration status indicated that refugees had the highest risk for subsequent specialized PTSD treatment [aHR 8.18; 95% CI:7.85–8.51], followed by non-refugee migrants [aHR: 3.09; 95% CI:3.0–3.19] and second-generation migrants [aHR 1.59; 95% CI:1.54–1.65] compared to the Swedish-born with Swedish-born parents.

**Table 2.** Hazard ratios (HR) with 95% confidence intervals (CIs) for post-traumatic stress disorder in the study population.

Covariates	Model 1^a^ HR (95% CI)	Model 2^b^ HR (95% CI)	Model 3^c^ HR (95% CI)	Model 4^d^ HR (95% CI)
Sex				
Male	1	1	1	1
Female	2.06 (2.02–2.11)	2.02 (1.97–2.06)	2.01 (1.96–2.05)	2.0 (1.96–2.05)
Age (in years)				
15–19	1	1	1	1
20–29	0.61 (0.59–0.63)	0.7 (0.65–0.75)	0.72 (0.67–0.78)	0.75 (0.7–0.8)
30–39	2.02 (1.95–2.09)	1.22 (1.13–1.32)	1.29 (1.19–1.39)	1.34 (1.24–1.45)
40–49	9.16 (8.61–9.74)	2.48 (2.26–2.73)	2.71 (2.47–2.98)	2.93 (2.66–3.22)
50–59	9.2 (5.93–14.26)	2.59 (1.66–4.03)	2.84 (1.82–4.44)	3.09 (1.98–4.81)
Education (in years)				
0–9	1	1	1	1
10–12	0.48 (0.46–0.49)	0.65 (0.63–0.68)	0.64 (0.62–0.67)	0.64 (0.62–0.67)
≥13	0.59 (0.57–0.62)	0.6 (0.57–0.63)	0.58 (0.55–0.61)	0.57 (0.55–0.6)
Missing	4.39 (4.25–4.54)	1.95 (1.88–2.03)	1.79 (1.73–1.86)	1.75 (1.68–1.81)
Family composition				
With partner no child	1	1	1	1
With partner and child	0.87 (0.82–0.9)	1.09 (1.03–1.16)	1.07 (1.0–1.13)	1.06 (0.99–1.12)
Single^e^ no child	0.47 (0.45–0.5)	1.1 (1.03–1.17)	1.09 (1.03–1.16)	1.09 (1.03–1.16)
Single with child	1.1 (1.03–1.18)	1.47 (1.37–1.58)	1.44 (1.34–1.55)	1.44 (1.34–1.54)
With parents, aged <20	0.71 (0.67–0.75)	0.95 (0.88–1.03)	0.93 (0.85–1.01)	0.91 (0.84–0.99)
Missing	3.26 (1.36–7.82)	16.69 (2.37–117.52)	17.63 (2.5–124.46)	17.7 (2.51–124.74)
Household income				
First quintile (lowest)	6.59 (6.16–7.04)	2.9 (2.7–3.11)	2.9 (2.71–3.12)	2.93 (2.73–3.14)
Second quintile	2.67 (2.49–2.87)	1.77 (1.65–1.91)	1.78 (1.65–1.92)	1.78 (1.65–1.92)
Third quintile	1.95 (1.81–2.1)	1.48 (1.37–1.59)	1.48 (1.37–1.6)	1.49 (1.38–1.6)
Fourth quintile	1.34 (1.24–1.46)	1.17 (1.08–1.27)	1.17 (1.08–1.27)	1.17 (1.08–1.27)
Fifth quintile (highest)	1	1	1	1
Missing	15.27 (5.76–40.52)	0.28 (0.03–3.02)	0.26 (0.02–2.82)	0.25 (0.02–2.78)
Living area^f^				
Large cities	1	1	1	1
Medium-sized towns	0.83 (0.81–0.83)	0.91 (0.88–0.93)	0.9 (0.88–0.93)	0.91 (0.88–0.93)
Small towns/rural area	0.8 (0.78–0.83)	0.93 (0.90–0.96)	0.93 (0.9–0.95)	0.93 (0.91–0.96)
Missing	3.4 (0.86–13.41)	0.78 (0.11–5.57)	0.78 (0.11–5.51)	0.78 (0.11–5.5)
Parental country of birth				
Both born in Sweden	1	—	—	—
At least one born outside Sweden	2.25 (2.19–2.32)	—	—	—
Missing	6.25 (6.09–6.42)	—	—	—
Migration status				
Non-migrants	1	1	1	1
Second generation migrants	1.79 (1.73–1.86)	1.63 (1.58–1.69)	1.61 (1.56–1.67)	1.59 (1.54–1.65)
Non-refugee migrants	4.38 (4.26–4.49)	2.92 (2.83–3.01)	3 (2.9–3.09)	3.09 (3.0–3.19)
Refugee migrants	11.38 (10.96–11.8)	7.79 (7.48–8.12)	7.98 (7.67–8.31)	8.18 (7.85–8.51)
Psychiatric comorbidity at baseline (reference “no”)				
Non-affective psychosis	22.06 (15.23–31.95)	—	1.9 (1.3–2.77)	2.14 (1.47–3.13)
Depressive disorders	23.21 (20.93–25.73)	—	4.88 (4.26–5.58)	4.51 (3.95–5.14)
Anxiety disorders other than PTSD	19.32 (17.5–21.32)	—	4.64 (4.08–5.27)	3.96 (3.5–4.49)
Alcohol use disorders	6.86 (5.92–7.94)	—	3.82 (3.28–4.45)	3.42 (2.94–3.99)
Other mental disorders	7.12 (6.61–7.64)	—	3.98 (3.66–4.34)	2.78 (2.55–3.03)
Suicide attempt	28.69 (22.97–35.83)	—	1.25 (0.98–1.6)	1.3 (1.02–1.65)
Any psychiatric	8.81 (8.33–9.32)	—	—	—
Somatic comorbidity (reference “no”)				
Circulatory disorders	3.86 (3.24–4.59)	—	—	1.9 (1.59–2.27)
Respiratory disorders	3.46 (3.26–3.66)	—	—	2.62 (2.46–2.8)
Diabetes	3.06 (2.43–3.87)	—	—	1.8 (1.42–2.28)
Musculoskeletal disorders	3.71 (3.47–3.97)	—	—	2.51 (2.33–2.69)

*Note: “—” means not included in the model.*

Abbreviation: PTSD, posttraumatic stress disorder.

a Model 1: Crude model.

b Model 2: Adjusted for sex, age, education, family composition, weighted household income, living area, parental country of birth, migration status.

c Model 3: Adjusted for model 2 and psychiatric disorders that are mentioned in the table.

d Model 4: full adjusted model – adjusted for model 3 and major somatic disorders that are mentioned in the table.

e Single includes divorced, separated or widowed.

f Large cities: municipalities with at least 200,000 inhabitants with at least 200,000 in the largest urban area (Stockholm, Gothenburg, and Malmo); Medium-sized cities: municipalities with at least 50,000 inhabitants with at least 40,000 in the largest urban area; Small cities/villages: municipalities with at least 15,000 inhabitants in the largest urban area.

Despite the overall low prevalence, psychiatric comorbidities were significantly associated with subsequent PTSD, managed at specialized care for PTSD, even after controlling for a number of potential confounders. Stepwise analyses indicated that the very high risk of PTSD in individuals with NAP or depressive disorders or suicide attempt in the crude model attenuated strongly after adjusting for the comorbid anxiety disorders. A further attenuation was observed following the inclusion of “other mental disorders” in the model. Association between common mental disorders and subsequent specialized PTSD treatment was the most prominent with around four-fold increased risk [depression-aHR 4.51; 95% CI:3.95–5.14; anxiety other than PTSD-aHR 3.96; 95% CI:3.5–4.49] (Table 2). Similar risks due to NAP and alcohol use disorders were [aHR 2.14; 95% CI:1.47–3.13] and [aHR 3.42; 95% CI: 2.94–3.99], respectively. Individuals with a history of suicide attempt were at more than 30% higher PTSD risk compared to those without such a history [aHR 1.3; 95% CI: 1.02–1.65].

Approximately 2–3% of the study population had a respiratory or musculoskeletal disorder at baseline. All four major somatic disorders were more prevalent in the PTSD population (Table 2). Respiratory [aHR 2.62; 95% CI: 2.46–2.8] and musculoskeletal [aHR 2.51; 95% CI: 2.33–2.69] disorders were associated with quite high specialized PTSD treatment risk followed by circulatory disorders [aHR 1.9; 95% CI: 1.59–2.27] and diabetes [aHR: 1.8; 95% CI:1.42–2.28].

## Discussion

### Main findings

In this cohort study of 4,673,764 individuals from across the whole of Sweden, 0.7% were treated for PTSD in specialized care services between 2006–2016. Incidence of specialized healthcare use due to PTSD nearly doubled from 2006 to 2016 and appears to be on an upward trajectory. We found that PTSD risk was associated with migration status, pre-existing psychiatric morbidity, low household income or education, female sex, older age, single parenthood, and urbanicity.

### Methodological consideration

To the best of our knowledge, this is the first-ever register-based study including an entire population that examines the incidence of specialized care for PTSD, and its association with sociodemographic and medical factors assessed pre-onset, thus avoiding recall bias. The Swedish register data are of good coverage and PTSD has been shown to have good validity in the patient register [7–15]. Additionally, given the social gradient in psychiatric morbidity [19, 20], using register data is likely to cover substantially more patients regardless of their socioeconomic position (SEP) which is a valuable advantage for the generalizability of our findings. However, the patient register contains information only from specialized care services and not from primary care. This can be considered as a strength as the outcome is likely to be more strictly defined, meaning that PTSD outcomes defined in our study are more likely to be valid. On the other hand, by not including primary care data, we might have missed PTSD patients with milder symptoms who were never referred to specialist care, which might lead to an underestimation of the risk estimates. Furthermore, some people with PTSD never present to primary care. Around 34% of the PTSD patients in our study were migrants, and this might be an underestimation given both a lower psychiatric healthcare utilization among migrants compared to the Swedish-born [21] and a higher risk of PTSD in this group. The low numbers of emigration and receiving specialized care for PTSD before 2006 seem low, however, given that over 60% of the study population were 15–19 years old at inclusion it is not surprising that very few had been treated at specialized care for PTSD before 2006 or emigrated from Sweden during the study period. Also, while we adjusted for potential confounding in our estimates, we cannot rule out residual confounding, for instance, looking at PTSD risk by birth country instead of migration status would provide more detailed and precise information. In this study, we controlled for pre-existing psychiatric comorbidity recorded before the start date of follow up, however, it is quite possible that some, for example, suicide attempt, could be perceived as a traumatic event. If depression is followed by suicide attempt, then it might mediate the effect from depression to PTSD. Distinguishing pre-, peri- and post-trauma factors will likely help our understanding of PTSD etiology and outcome, but such information was not available in our dataset, and to collect such data by survey from the entire population aged 15–64 years was not in the scope of this work. Nevertheless, given the explorative nature of our work, and the fact that other psychiatric diagnoses identified after the start of follow-up are not adjusted for, we might have underestimated the true association between other disorders and subsequent PTSD.

Utilization of specialized healthcare for PTSD nearly doubled during 2006–2016. This could reflect the overall increase in psychiatric care utilization over these years in Sweden [22] decreasing the number of individuals with unmet needs of care [22]. Additionally in 2010, that is within the timeframe of follow up of this study, there were new national guidelines for the care of patients with depression or anxiety disorders (including PTSD) including guidelines on screening tools and this could have increased detection [23]. Also, until 2015, Sweden has been one of the EU countries to accept most refugees (who have a high PTSD risk, see below) on a per capita basis, granting more refugee applications than any other high-income country, leading to a change in its demographics during these years. During the years of follow up, there was a change from the diagnostic and statistical manual of mental disorders (DSM)-IV to DSM-5, however, we have no reason to believe that this is the reason for the increase [15].

We found age and sex variations in PTSD risk in our study population, as females and older age groups were at a higher risk for specialized care use for PTSD compared to males and younger age groups, respectively. These findings are in line with previous research [24, 25].

Our results indicated an inverse dose–response association between education and PTSD risk. Analogous finding, although with regional variations in trauma type, was reported from the WMH surveys [25, 26], and might be explained by a higher likelihood of exposure to traumatic events in lower educated individuals [25, 26]. The individuals with missing information on education had the highest hazards of specialized PTSD treatment in our population. The majority in this “missing” group were migrants either with low education or whose educational level from their home country was not validated in the Swedish system [5, 26]. Similar to education, an inverse dose–response association between weighted household income and specialized care for PTSD risk was observed, consistent with the findings that traumatic and other stressful events are more common among those with low SEP [25].

It is worth mentioning that the association between low education level, low household income, and high PTSD risk is indicative of a social gradient for PTSD, as commonly observed in psychiatry [19, 20], although previously inconsistently reported for PTSD [27]. Our study was able to capture evidence of such a gradient due to two facts: first, Sweden has publicly funded universal health care which meets the need of those with low SEP [20], and second, we used register-based data which includes everyone, regardless of their SEP.

Our results demonstrate that those living alone have an increased risk of subsequent specialized care for PTSD, and that single parents were at the highest risk. Previous studies have reported an elevated PTSD risk among unmarried persons [25]; however, this is the first study ever to include such detailed information on parenthood.

We observed a lower risk for specialized PTSD care among people living in medium/small towns or villages compared to city residents. Frans et al. [5], in 2005, also reported a higher prevalence of lifetime PTSD and traumatic events among urban dwellers than in rural in a Swedish setting. Given the well-established evidence that urbanicity is a risk indicator for psychosis [28], and the relationship between PTSD and psychosis, [29] this finding warrants further research.

Migration is one of the most important risk indicators for PTSD, and even more relevant due to increasing migration in the recent past. The stressful experiences of forced resettlement among asylum seekers and refugees may impact their mental health and increase the risk of PTSD [30–32]. The process of resettlement to the host country is different between refugee and non-refugee migrants. We found large discrepancies in PTSD risk across different migrant groups. Although all refugees in this population had been granted asylum and were granted residence permits, they had a higher risk of specialized care for PTSD than non-refugee migrants (eight-folds vs. three-folds, respectively). Second-generation migrants had around one and a half times higher risk compared to the Swedish-born, which was substantially lower than that of first-generation migrants. Parental immigration has been associated with an increased risk of traumatic experiences in the family and the child’s risk of developing PTSD following adverse events [33, 34].

The most prevalent comorbidities with PTSD are reported to be mood, anxiety, and substance use disorders [1, 24, 35], which is similar to our finding. Pre-existing depression may increase a person’s propensity to the PTSD-inducing effects of traumatic events [35], as well as increasing risk of exposure to traumatic events [36]. There exists a hypothesis of reciprocity between depression and PTSD, whereby depression increases the risk of developing PTSD, and be a consequence, or be more severe following the onset of PTSD [37]; however, testing the latter was not within the scope of this paper.

Alcohol use disorders were associated with a greater than three-fold increase in PTSD risk in our study population. A recent Canadian study reported a nearly 80% increased PTSD risk among people with alcohol dependence [38]. The bidirectional association and temporality of alcohol use disorder and PTSD were tested by Berenz et al. [39] in 2017 using data from the U.S. National Survey on Alcohol and Related Conditions. The authors reported that initial alcohol use disorder was associated with greater likelihood of subsequent PTSD, and the association was stronger among females [39]. It is not unlikely that alcohol use disorder increases the exposure to traumatic events, for example, interpersonal violence, hence heightening PTSD risk.

Pre-existing NAP, although nine times higher among PTSD patients than in the general population, was not very common in our study population. However, it heightened the risk of specialized care for PTSD by more than two-fold compared to those without NAP. While the majority of the studies looking at psychosis and PTSD did not distinguish between affective and non-affective psychoses, or focused on first-episode psychosis [40], our study reports NAP as a risk indicator for PTSD [41]. Evidence suggests a possible overlap between negative symptoms of psychosis and avoidance symptoms in PTSD, as well as positive psychosis symptoms and PTSD re-living [29], which could be an explanation for higher PTSD levels among patients with NAP. However, further research is needed to understand the pathways between NAP and PTSD.

Common genetic risk factors for different psychiatric disorders including mood disorders [37], alcohol abuse [39], psychosis [42], and PTSD may help explain the higher PTSD risk in patients with psychiatric comorbidity.

Respiratory and musculoskeletal disorders were more common than circulatory disorders or diabetes in our study population. The association between circulatory disorder and PTSD has been well researched [43], and the known high risk of PTSD among cardiovascular patients is in line with our results. In our study population, the risk of specialized care for PTSD was nearly two times higher among people with diabetes compared to those without. PTSD has been shown to elevate the risk for metabolic disorders including diabetes [44], however, evidence on whether the association is reciprocal or whether diabetes mediates the risk from any other factors is lacking.

## Conclusion

The incidence of specialized healthcare utilization for PTSD nearly doubled between 2006 and 2016, possibly due to demographic changes, with an increase in refugees who are at higher risk for PTSD but also due to better detection of PTSD in the population. There are effective treatments for PTSD, and the faster this is provided the less the suffering and risk of additional negative consequences. Our work provides evidence on groups that are likely to have greater need for specialized care for PTSD, including refugee migrants and those with other psychiatric disorders or who are socioeconomically disadvantaged. Future research should focus on how best to deliver evidence-based therapy for PTSD to those most in need of it.

## Data Availability

The register data used in this study contain sensitive information at an individual level and therefore, are not publicly available due to confidentiality. However, in special situation, access to the data can be directly requested to data provider. Codes can be made available upon request.
